# Engineering super- and sub-radiant hybrid plasmons in a tunable graphene frame-heptamer metasurface

**DOI:** 10.1515/nanoph-2025-0300

**Published:** 2025-10-27

**Authors:** Jiayi Gui, Na Chen, Hanchao Teng, Zhuoxin Xue, Shuang Xi, Chengyu Jiang, Shenghan Zhou, Hualong Zhu, Hai Hu

**Affiliations:** Laboratory of Nanophotonic Materials and Devices, Laboratory of Standardization and Measurement for Nanotechnology, 85389 National Center for Nanoscience and Technology, Beijing 100190, P.R. China; Center of Materials Science and Optoelectronics Engineering, University of Chinese Academy of Sciences, Beijing 100049, P.R. China; School of Materials Science and Engineering, Shanghai Jiao Tong University, Shanghai 200240, P.R. China

**Keywords:** graphene metasurface, hybrid plasmons, super- and sub-radiance, polarization control, electrostatic tuning

## Abstract

Controlling near-field electromagnetic interactions is central to tailoring optical responses in plasmonic systems. However, the static nature of conventional noble metal nanostructures limits their application in active photonic devices. In this work, we design and experimentally demonstrate a composite graphene metasurface, composed of an octagonal frame coupled to a central heptamer disk, that enables multidimensional and active control over hybrid plasmons. The observed rich spectral features originate from hybridization between the dipolar and higher-order modes of the frame and the collective resonances of the heptamer. We show that the polarization of incident light serves as an effective control parameter for engineering the radiative properties of these modes. By varying the polarization angle, specific resonances can be selectively driven into super-radiant states with enhanced radiation or sub-radiant states with suppressed emission. In parallel, electrostatic gating provides a second, independent tuning mechanism that enables wide, continuous, and robust spectral modulation, in excellent agreement with theoretical predictions. The combined use of structural design, polarization control, and electrical tuning transforms a static metasurface into a dynamically reconfigurable platform. This dual control over both resonance frequency and radiative coupling offers a comprehensive toolkit for on-demand manipulation of light–matter interactions, paving the way for advanced optical modulators, reconfigurable filters, and tunable sensing technologies.

## 1 Introduction

The ability to manipulate light–matter interactions at the nanoscale is a cornerstone of modern optics and condensed matter physics. A particularly powerful platform for this is the “plasmonic molecule”, an artificial nanostructure formed by the near-field coupling of individual plasmonic resonators [1], [2], [3], [4]. These complex oligomers can generate novel optical phenomena, such as Fano resonances, plasmon-induced transparency, complex near-field multipolar modes, and enhanced nonlinear optical effects, that are absent in their constituent parts [5], [6], [7], [8]. Leveraging their mature fabrication processes and excellent intrinsic plasmonic properties, noble metals such as gold and silver [9] dominated the early work in this field. However, their static dielectric properties present a critical bottleneck: once fabricated, their optical response is fixed [10], [11], severely limiting their application in active devices and, crucially, prevents dynamic control over the radiative properties of coupled modes.

To overcome this limitation, two-dimensional materials, with graphene as the leading example, offer a transformative solution [12], [13], [14]. Graphene supports strongly confined plasmons from the mid-infrared to the terahertz range, and more importantly, its optical conductivity can be continuously and dynamically tuned via an external gate voltage, providing the key to creating active plasmonic coupled devices [15], [16], [17], [18]. Furthermore, operating in the mid-infrared relaxes the stringent demands on fabrication precision that challenge visible-light plasmonics, making the creation of complex, high-performance devices more feasible [19], [20], [21], [22].

In this work, we leverage these advantages to demonstrate the active engineering of radiative modes in a complex plasmonic system. We have designed and fabricated a composite graphene metasurface composed of an octagonal frame coupled to a central heptamer disk cluster. The intricate spectral response of this structure arises from strong hybridization between the fundamental and higher-order modes of the frame and the collective dipole mode of the heptamer. Furthermore, by carefully selecting the polarization of incident light, we selectively excite specific modes, forcing them into pronounced super-radiant or sub-radiant regimes, thereby achieving multi-dimensional, active control over the radiative character of these hybridized states. Concurrently, we demonstrate that the resonance frequencies of these engineered states can be continuously and robustly shifted via electrostatic gating. This synergistic approach, integrating structural design with polarization and electrical control, transforms a static metasurface into a dynamically reconfigurable platform for manipulating radiative damping and energy pathways at the nanoscale, opening new avenues for dynamically reconfigurable devices such as advanced optical sensors, modulators, and programmable photonic elements [23], [24], [25], [26], [27], [28].

## 2 Results and discussion

**Figure 1:**
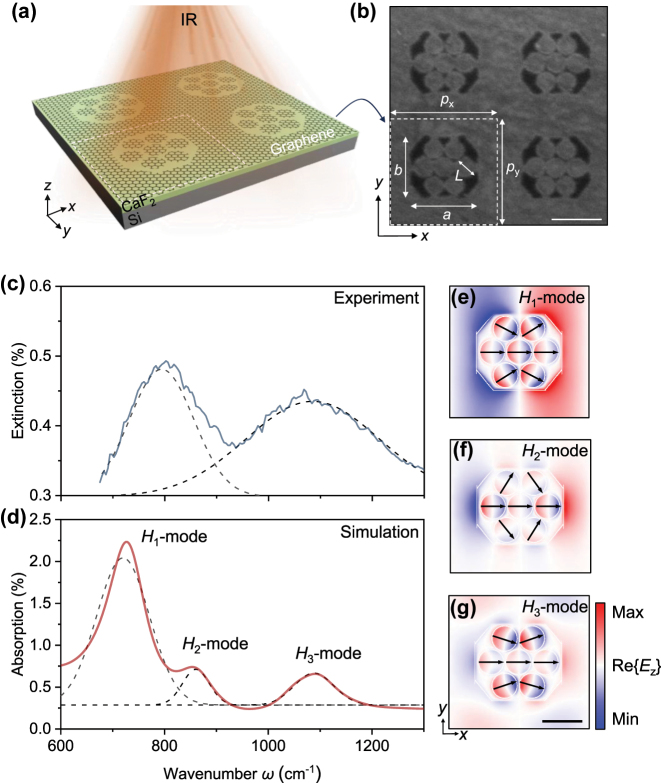
Structure and hybrid plasmons of the graphene metasurface. (a) Schematic of the metasurface, consisting of a periodically patterned graphene layer on a CaF_2_/Si substrate, a configuration that enables electrostatic gating. (b) Scanning electron microscope (SEM) image of the fabricated structure. The unit cell has periods of *p*_*x*_ = 730 nm and *p*_*y*_ = 690 nm. The octagonal frame has side lengths of *a* = 450 nm and *b* = 400 nm, and encloses a central heptamer of seven disks, each with a diameter *L* = 130 nm. The dimensions are optimized to account for both fabrication tolerance and the desired spectral peak position (Supplementary Figure 1). Narrow (∼5 nm) nanobridges connect the elements to ensure uniform carrier concentration across the structure. Scale bar represents 400 nm. (c) Experimental extinction spectrum (solid curve) for a graphene Fermi energy of 0.6 eV. (d) Corresponding simulated absorption spectrum (solid curve). Lorentzian fits (dashed curves) on both spectra reveal that the two experimentally observed peaks correspond to the simulated hybrid modes *H*_2_ and *H*_3_. The simulation predicts a third mode, *H*_1_, at a lower wavenumber outside the experimental detection range. (e–g) Simulated near-field distributions of the real part of the out-of-plane electric field (Re{*E*_*z*_}), plotted 10 nm above the graphene layer, for the three hybrid modes: (e) *H*_1_, (f) *H*_2_, and (g) *H*_3_. Scale bar is 200 nm for all panels.

To investigate plasmonic coupling in complex geometries, we designed and fabricated a composite metasurface featuring a graphene octagonal frame coupled to an internal heptamer disk cluster (Figure 1a). The entire structure is fabricated on a CaF_2_/Si heterostructure, which serves as a back gate to dynamically tune the carrier concentration in the graphene [29]. As shown in the SEM image in Figure 1b, a key design feature is the inclusion of narrow (∼5 nm) conductive nanobridges connecting the individual graphene elements. These bridges ensure a uniform carrier concentration across the unit cell while being small enough to avoid the significant impact on the overall spectral line shape and peak position that a larger structure would cause (Supplementary Figures 2 and 3).

Figure 1c presents the experimental extinction spectrum of the metasurface, measured at a graphene Fermi energy (*E*_F_) of 0.6 eV. A Lorentzian fit to the data (dashed curve) reveals two prominent resonance peaks centered at approximately 800 cm^−1^ and 1,100 cm^−1^. To gain deeper insight into the origin of these resonances, we performed corresponding numerical simulations, with the calculated absorption spectrum shown in Figure 1d. The simulation qualitatively matches the experimental result but predicts three distinct resonance peaks, which are labelled as the hybridized modes *H*_1_, *H*_2_, and *H*_3_. The resonant frequencies of *H*_2_ and *H*_3_ are consistent with the experimental results, but the *H*_1_ mode could not be effectively observed due to the limitations of the measurement window. We also note that the full width at half maximum (FWHM) and peak intensity of the experimental peaks differ from the simulation results, a common effect attributed to unavoidable nanoscale imperfections during fabrication [11], which will be further explained in the subsequent discussion. To identify the physical nature of each resonance and visualize the mode coupling, we simulated the near-field electric field distributions (Re{*E*_*z*_}) corresponding to each peak, as shown in Figure 1e–g. The spatial maps reveal starkly different characteristics for each mode. The electric field of the *H*_1_ mode (Figure 1e) is highly localized on the octagonal frame, exhibiting the clear signature of a dipolar resonance [30], [31]. In contrast, the electric field of the *H*_2_ (Figure 1f) and *H*_3_ (Figure 1g) modes is predominantly confined within the inner heptamer cluster, displaying more complex, higher-order collective oscillation patterns [32]. This analysis provides a direct link between the macroscopic spectral features and the microscopic oscillations, establishing the distinct character of the three fundamental hybrid modes, which form the basis for the subsequent engineering of super- and sub-radiant modes.

**Figure 2:**
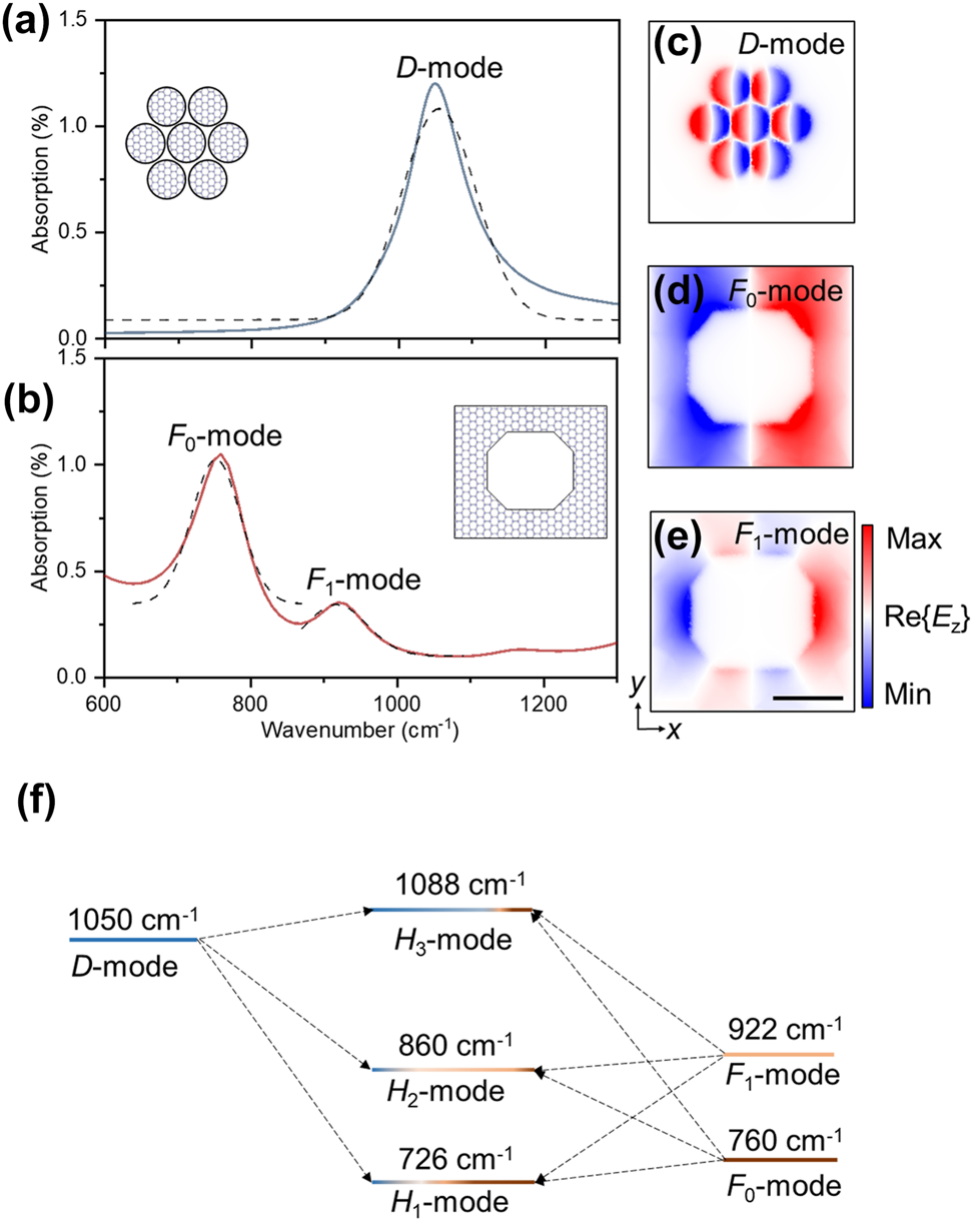
Analysis of hybrid modes and plasmonic coupling system. (a) Simulated absorption spectra and corresponding near-field distributions (Re{*E*_*z*_}) for the individual, uncoupled components of the metasurface. The isolated heptamer disk cluster supports a single collective dipole resonance, termed the *D*-mode, at 1,050 cm^−1^. (b) The isolated octagonal frame exhibits two distinct resonances: a fundamental dipole mode (*F*_0_-mode) at 760 cm^−1^ and a higher-order mode (*F*_1_-mode) at 922 cm^−1^. (c–e) Near-field distributions for the “bare” eigenmodes: (c) the collective *D*-mode of the heptamer, (d) the dipolar *F*_0_-mode of the frame, and (e) the higher-order *F*_1_-mode of the frame. Scale bar is 200 nm. (f) Energy-level diagram illustrating the mode hybridization. The eigenmodes of the isolated heptamer (*D*-mode, left) and frame (*F*_0_ and *F*_1_-modes, right) act as the “bare” states. When brought together, near-field coupling causes these bare modes to interact and hybridize, forming three new modes for the composite system (*H*_1_, *H*_2_, *H*_3_, center). The mixed-color bars schematically represent the contribution of each bare mode to the final hybrid modes.

To elucidate the physical origins of the three hybrid modes observed in Figure 1, we employ a theoretical framework based on coupling, the core concept of which is to analyze the response of the composite structure by characterizing the individual plasmonic eigenmodes of its uncoupled components [33]. As shown in Figure 2a, the isolated heptamer disk cluster exhibits a single, strong resonance at approximately 1,050 cm^−1^. Its near-field distribution (Figure 2c) reveals a collective, in-phase dipolar oscillation, which we designate as the *D*-mode [34]. Conversely, the isolated octagonal frame supports two primary resonances (Figure 2b): a strong, fundamental dipole absorption peak at ∼760 cm^−1^, which we term the *F*_0_-mode (Figure 2d), and a weaker, higher-order absorption peak at ∼922 cm^−1^, termed the *F*_1_-mode. The significantly lower absorption intensity of the *F*_1_-mode is a direct consequence of its multipolar nature. Unlike the *F*_0_-mode, which possesses a large net dipole moment, the field symmetry of the *F*_1_-mode (Figure 2e) results in a very small net dipole moment, thereby suppressing its far-field coupling and minimizing its radiative damping rate [28].

When these individual components are brought into proximity within the composite metasurface, their “bare” eigenmodes (*D*, *F*_0_, and *F*_1_) interact via near-field coupling to form new, hybridized system-level modes, a process analogous to molecular orbital theory [35], [36]. The interaction among the three individual modes leads to the formation of three hybridized resonances, labeled *H*_1_, *H*_2_, and *H*_3_, as observed in the full-system simulation (Figure 1d). The hybridization diagram in Figure 2f illustrates this energy renormalization process [37]. The strong coupling between the two primary dipolar modes, *D* and *F*_0_, leads to a significant energy splitting, forming a lower-energy “bonding-like” state (*H*_1_) at 726 cm^−1^ and a higher-energy “anti-bonding-like” state (*H*_3_) at 1,088 cm^−1^. Analysis of the near-field plots of the hybrid modes confirms this picture: the energy of the *H*_1_ mode is primarily concentrated on the frame, inheriting the character of the *F*_0_-mode, while the *H*_3_ mode energy is localized within the heptamer, indicating it is dominated by the *D*-mode. The intermediate mode *H*_2_, located at 860 cm^−1^, results from a more complex interaction involving the higher-order *F*_1_ mode of the frame, as evidenced by its distinct near-field pattern [34].

This hybridization model not only explains the spectral features but also offers a physical framework for engineering the response of the system. The energy splitting between the bonding (*H*_1_) and anti-bonding (*H*_3_) modes is highly sensitive to the coupling strength [34], which is governed by geometric parameters like the spacing between elements (Supplementary Figure 4). This sensitivity allows for the rational design of spectral features and indicates that such structures hold significant potential for high-sensitivity optical sensing, where minor environmental perturbations can be transduced into large, measurable spectral shifts [27], [38], [39], [40].

**Figure 3:**
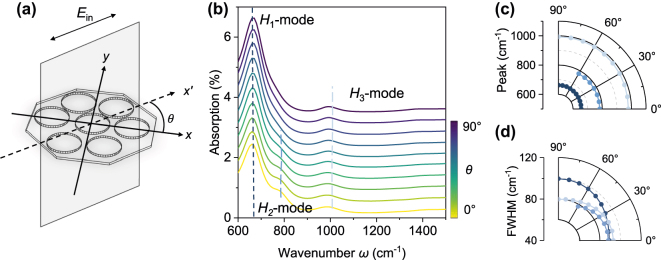
Engineering super- and sub-radiant states via polarization manipulation. (a) Schematic defining the polarization angle, *θ*, between the incident electric field (*E*_in_, aligned with the *x*′-axis) and the primary axis (*x*-axis) of the metasurface structure. All simulations are performed with a graphene Fermi energy of 0.5 eV. (b) Polarization-dependent absorption spectra as *θ* is varied from 0° to 90°. While the resonance frequencies of the three hybrid modes (*H*_1_, *H*_2_, *H*_3_) remain stable, the absorption intensity of the *H*_2_-mode is dramatically suppressed as *θ* approaches 90°. This behavior is a hallmark of a transition to a sub-radiant, caused by destructive interference between coupled oscillators. (c) Polar plot of the resonance frequencies, confirming their stability across the full range of polarization angles. (d) Polar plot of the full width at half maximum (FWHM), which reflects the radiative damping rate of each mode. The significant broadening of the *H*_1_ mode FWHM near *θ* = 90° indicates enhanced radiative coupling, characteristic of a super-radiant mode.

Given that the composite structure we designed lacks rotational symmetry [41], [42], its radiative response should logically exhibit a dependence on the polarization of incident light, providing an additional degree of freedom for tuning in device design. Here, we systematically investigated the evolution of the hybrid modes under various incident polarization angles, *θ* (Figure 3a and Supplementary Figure 5). Figure 3b presents the simulated absorption spectra as *θ* is continuously varied from 0° to 90°, with the graphene *E*_F_ fixed at 0.5 eV. A striking feature, quantified in Figure 3c, is that the resonance frequencies of all three hybrid modes (*H*_1_, *H*_2_, and *H*_3_) remain remarkably stable across the entire polarization range. It should be noted that this stability is not a true stability but rather a “metastable state”. This phenomenon originates from the coincidental alignment of the resonance peak positions for the 0° and 90° polarization responses, which results in the peak positions of the coupled response showing almost no shift as the polarization varies between these angles (Supplementary Figure 6).

In stark contrast to the stable frequencies, the absorption intensities exhibit strong and distinct polarization dependencies. Most notably, the intensity of the *H*_2_ mode monotonically decreases as *θ* increases until it is almost completely suppressed at *θ* = 90°, a direct signature of the system being driven into a sub-radiant (or “quasi-dark”) mode [43], [44], which originates from a significant phase shift developing between the resonance phases of the seven-disk cluster and the octagonal frame (indicated by the black solid line in Supplementary Figure 7), resulting in destructive interference that cancels the two electromagnetic modes and reduces the system’s net dipole moment to a value that is very small, or even zero. Consequently, its ability to couple with the external light field is substantially suppressed, rendering the mode unable to easily absorb external energy or readily radiate its own energy away. Although its far-field radiative response is suppressed, the sub-radiant mode produces a significant field enhancement due to its extremely strong near-field confinement (Supplementary Figure 8), providing a highly promising technological path for achieving ultrasensitive, label-free molecular detection.

In contrast, the *H*_1_ mode exhibits super-radiant characteristics under specific polarizations, with its FWHM showing significant broadening at *θ* = 90°, indicating that its constituent components achieve an in-phase or nearly in-phase coupling under this polarization. Their dipole moments undergo constructive interference, forming an equivalent “super-dipole moment”, which greatly enhances the coupling between the mode and the external light field, leading to a significant increase in radiative damping, which in turn manifests spectrally as a broadening of the linewidth [28], [45]. The ability to utilize polarization-selective excitation of modes with different radiative properties expands the possibilities for achieving active and fine-grained control over energy flow and localized field distributions at the sub-wavelength scale, offering a new approach for designing polarization-multiplexed optical switches and modulators [46], [47].

**Figure 4:**
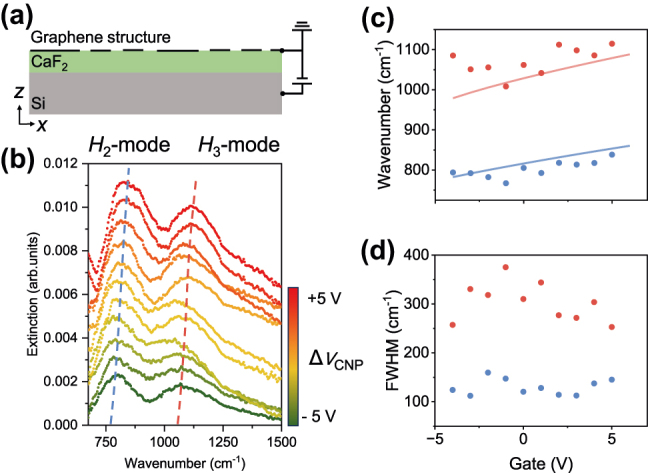
Electrical tuning of the hybrid plasmonic devices. (a) Schematic of the back-gated devices, where an applied voltage modulates the carrier concentration and the Fermi energy (*E*_F_) in the graphene metasurface. (b) Experimental extinction spectra as a function of the gate voltage relative to the charge neutrality point (Δ*V*_CNP_). As *E*_F_ is increased, the hybrid modes *H*_2_ (blue dashed lines) and *H*_3_ (red dashed lines) exhibit a clear and continuous blueshift. (c) Resonance frequencies of the *H*_2_ (blue) and *H*_3_ (red) modes plotted as a function of gate voltage. The experimental data (scattered dots) show excellent agreement with theoretical simulations (solid lines), confirming the robust electrical tunability of the devices. (d) Extracted FWHM for both modes. The linewidths remain relatively stable across a wide tuning range, indicating that the mode lifetimes are robust and largely independent of the carrier concentration.

Having established polarization as a powerful tool for engineering the radiative character of the hybrid modes, we further demonstrate the second, complementary dimension of control: active spectral tuning via electrostatic gating [15]. This capability, a core advantage of graphene plasmonics, is experimentally realized using the back-gated device architecture shown in Figure 4a. The applied gate voltage (Δ*V*_CNP_) directly modulates the Fermi energy of graphene (*E*_F_), enabling continuous tuning of the plasmon resonances.

The experimental results, presented in the series of extinction spectra in Figure 4b, unequivocally show this dynamic tuning capability. Leveraging the electric double-layer effect with a CaF_2_ gate [13], both *H*_2_ and *H*_3_ modes demonstrate a notable and continuous blueshift under a mere 10 V change in gate voltage. This wide-range tunability is quantitatively captured in Figure 4c, where the extracted experimental peak positions (scattered dots) show remarkable agreement with our theoretical model (solid lines and Supplementary Figure 9). The ability to achieve wide-range tuning at a low driving voltage shows great potential for future device applications. However, the device’s performance is currently limited by the quality and small dielectric function of the CaF_2_, which leads to a significantly increased risk of breakdown at excessive gate voltages. Therefore, we only demonstrate the modulation from –5 V to +5 V in this work.

Crucially, this impressive spectral tunability does not come at the cost of performance stability. As plotted in Figure 4d, the FWHM of both resonances remains relatively constant throughout the tuning process, indicating that the intrinsic damping rates of the hybrid modes are robust and largely unaffected by the change in carrier concentration, ensuring a predictable and reliable optical response during dynamic operation. It should be noted that, compared to a traditional noble metal such as gold and silver, graphene exhibits stronger near-field coupling. This pronounced field confinement amplifies the effects of subtle geometric imperfections, particularly the significant edge roughness near the nanobridges, which induces considerable inhomogeneous broadening and degrades the mode’s quality factor (Supplementary Figure 10). This largely accounts for the significant discrepancy in the FWHM compared to simulations (Supplementary Figure 11). Therefore, efforts to mitigate these losses through improvements in graphene quality and optimized device architectures constitute a critical avenue for future research.

The robust, wide-range, and continuous electrical tunability demonstrated by this graphene composite metasurface signals its immense potential in the field of dynamic optical devices. Its core advantage lies in a unique capability for decoupled dual-parameter control, a distinction that is particularly pronounced when compared to structurally identical gold arrays. Specifically, for gold-based devices, constrained by their static permittivity, only the resonance linewidth can be tuned under a 0°–90° polarization sweep, while the resonance frequency remains fixed. In contrast, graphene-based devices can modulate the *E*_F_ via gate voltage to achieve a resonance frequency shift of several hundred cm^−1^ without altering the linewidth. By combining this electrical control with the responses derived from mode hybridization and polarization selection, it is possible to construct a multi-degree-of-freedom synergistic control system. This would enable fine-grained, active manipulation of the light field in dimensions such as frequency, intensity, and polarization.

## 3 Conclusions

In conclusion, we have designed, fabricated, and systematically demonstrated a powerful strategy for the multi-dimensional, active control of hybrid plasmons within a composite graphene metasurface. By combining experiments and simulations, we reveal that the rich spectral landscape of the structure arises from the near-field hybridization of the plasmonic eigenmodes of the constituent frame and heptamer. Importantly, two independent and complementary control mechanisms have been established. One involves using the incident polarization to tailor the radiative properties of the system by selectively exciting pronounced super-radiant and sub-radiant modes. The other employs electrostatic gating to achieve wide, continuous, and robust tuning of the corresponding spectral positions.

This work advances beyond the passive and static nature of conventional plasmonic systems by integrating three hierarchical levels of control: structural engineering, polarization selectivity, and electrical tunability, all within a single dynamically reconfigurable platform. The capacity to independently tune both the spectral position of a mode and its radiative coupling strength offers a comprehensive toolkit for precise manipulation of light–matter interactions. The design strategy and control approach demonstrated here are broadly applicable and not limited to the specific geometry explored, opening pathways toward a new generation of intelligent and programmable optical components. Such reconfigurable metasurfaces present significant potential for future optoelectronic technologies, including high-speed spatial light modulators, multifunctional sensors with tunable sensitivity, reconfigurable optical filters, and advanced platforms for on-chip nonlinear optics and quantum information processing.

## 4 Methods

### 4.1 Graphene device fabrication

Graphene synthesized via chemical vapor deposition was initially transferred onto a substrate consisting of 300 nm SiO_2_ atop a 500 μm Si base using a standard wet-transfer technique. A 120-nm-thick layer of poly (methyl methacrylate) (PMMA, 950K) was then spin-coated onto the graphene surface. Metasurface arrays were subsequently defined in the graphene through electron beam lithography (Vistec 5000+ES, Germany), followed by oxygen plasma etching conducted at 5 Pa pressure and 80 W power for 10 s (SENTECH, Germany).

A second electron-beam lithography step, in conjunction with electron beam evaporation (OHMIKER-50B, Taiwan), was employed to deposit Cr/Au (5 nm/60 nm) electrodes. A CaF_2_ thin film was deposited at 100 °C under high vacuum conditions (∼10^−6^ Torr) with a growth rate of 0.5 Å/s. Finally, the graphene device originally on the SiO_2_ substrate was transferred onto the CaF_2_/Si support and annealed at 200 °C for 5 h to improve interface quality.

### 4.2 Characterization of graphene plasmonic devices

The structural features and thickness profiles of the graphene metasurface were examined using scanning electron microscopy (SEM, Hitachi S-4800) and atomic force microscopy (AFM, Neaspec s-SNOM). Raman spectroscopy (Horiba Jobin Yvon LabRAM HR800) with a 514 nm laser was employed to evaluate the crystalline quality and assess the defect levels in the graphene metasurface. The electronic performance of the devices was characterized using a semiconductor parameter analyzer (Agilent 4294A).

### 4.3 FTIR spectroscopy measurements

All spectroscopic measurements were performed using a commercial Fourier-transform infrared (FTIR) system, consisting of a Bruker Hyperion 2000 microscope coupled to a Vertex 70V spectrometer, which provided the broadband illumination for the experiments. The final extinction spectra (*η*) presented in this work were calculated according to the following relation: $\eta =1-T_{V_{\text{g}}}/T_{V_{\text{CNP}}}$ , where $T_{V_{\text{g}}}$ is the transmission spectrum through the doped device at a given gate voltage, and $T_{V_{\text{CNP}}}$ is the transmission spectrum of the device at its charge neutrality point.

### 4.4 Theoretical modeling and simulation

To complement our experimental findings, we performed electromagnetic simulations using the commercial finite-element solver, COMSOL Multiphysics (RF module). Within the simulations, the optical conductivity of graphene was modeled using the Kubo formula. All geometric parameters for the nanostructures were set to be consistent with our SEM imaging data. To accurately replicate the experimental conditions, we applied periodic boundary conditions to the unit cell, with the incident light defined as a normally incident plane wave. The total absorbance (*A*) was subsequently calculated from the simulated transmittance (*T*) and reflectance (*R*) via the relation *A* = 1 − *T* − *R.* For the near-field distributions shown in Figures 2 and 3, we extracted the electric field maps on a plane located 10 nm above the graphene surface.

## Supplementary Material

Supplementary Material Details
