# Supplementary material for: Engineering super- and sub-radiant hybrid plasmons in a tunable graphene frame-heptamer metasurface
Source: Nanophotonics. 2025 Oct 27;14(27):5059–67. doi: 10.1515/nanoph-2025-0300 (PMC12717923; doi:10.1515/nanoph-2025-0300)
Supplement: Supplementary file 1 — Supplementary Material Details [file j_nanoph-2025-0300_suppl_001.pdf]

Supplementary Information for

"Engineering super- and sub-radiant hybrid plasmons in a tunable graphene frame-heptamer metasurface"

This PDF file includes:

Supplementary Figure 1. Influence of structure dimensions.
Supplementary Figure 2. Influence of conductive nanobridges on optical response of isolated heptamer disks.
Supplementary Figure 3. Absorption spectra of conductive nanobridges with different widths.
Supplementary Figure 4. Inter-disk spacing tuning in heptamer disks.
Supplementary Figure 5. Top-down view of the metasurface structure.
Supplementary Figure 6. Optical response of graphene plasmonic octagonal frame and heptamer disk under incident polarization angles of 90°.
Supplementary Figure 7. Optical response of a graphene plasmonic octagonal frame with a heptamer disk under incident polarization angles of 0° and 90°.
Supplementary Figure 8. Simulated electric near-field intensity enhancement.
Supplementary Figure 9. Tuning graphene plasmons with graphene Fermi energy.
Supplementary Figure 10. Comparison of experimental and simulated linewidths.
Supplementary Figure 11. Simulated absorption spectra for graphene plasmons at different carrier mobilities.

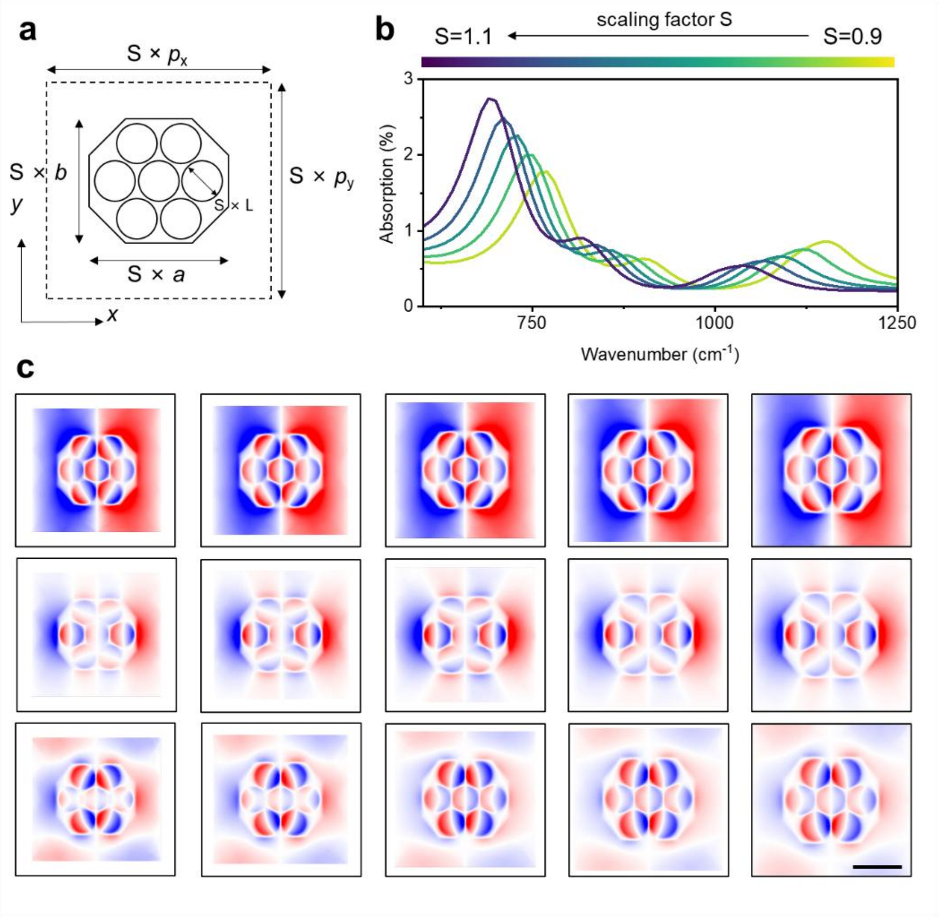

Supplementary Figure 1. Influence of structure dimensions. (a) The structure size is controlled by scaling the unit cell lateral dimensions with a factor *S*. The unit cell has periods of p_x = 730 nm and p_y = 690 nm. The octagonal frame has side lengths of *a* = 450 nm and *b* = 400 nm, and encloses a central heptamer of seven disks, each with a diameter *L* = 130 nm. *S* ranges from 0.9 to 1.1 in increments of 0.05. **(b)** Simulated absorption spectra for a graphene Fermi energy of 0.6 eV. The simulation predicts three modes, H_1, H_2 and H_3. **(c)** Simulated near-field distributions of the real part of the out-of-plane electric field (Re{E_z}), plotted 10 nm above the graphene layer, for the three hybrid modes with *S* increasing from left (0.9) to right (1.1): H_1 (top), H_2 (middle), and H_3 (bottom). Scale bar is 200 nm for all panels.

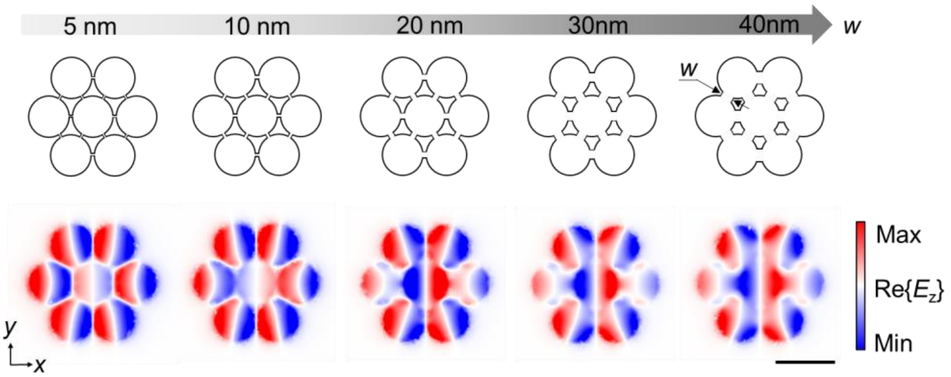

Supplementary Figure 2. Influence of conductive nanobridges on optical response of isolated heptamer disks. Schematic of the isolated graphene heptamer disk array with conductive nanobridges (*w* = 5, 10, 20, 30, 40 nm). Schematic (top) and corresponding spatial distribution of the real part of the electric field *z*-component (Re{E_z}, bottom) for the fundamental *D*-mode at 10 nm above the graphene surface. When the width of the nanobridges is less than 10 nm, the electric field distribution shows remarkable consistency, exhibiting distinct dipole oscillation behavior. As *w* increases, the electric field intensity gradually contracts towards the edge of the graphene disks, demonstrating the coupling effect of the nanobridges on the graphene disks. The scale bar represents 200 nm for all panels.

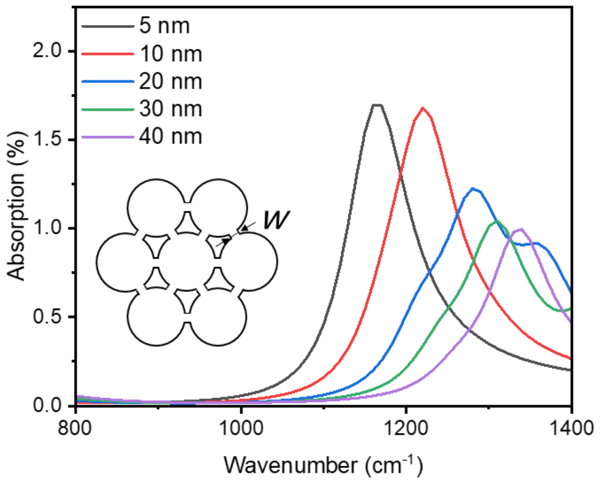

Supplementary Figure 3. Absorption spectra of conductive nanobridges with different widths. Simulated absorption spectra of the graphene heptamer disk array with varying conductive nanobridge widths (*w* = 5, 10, 20, 30, 40 nm).

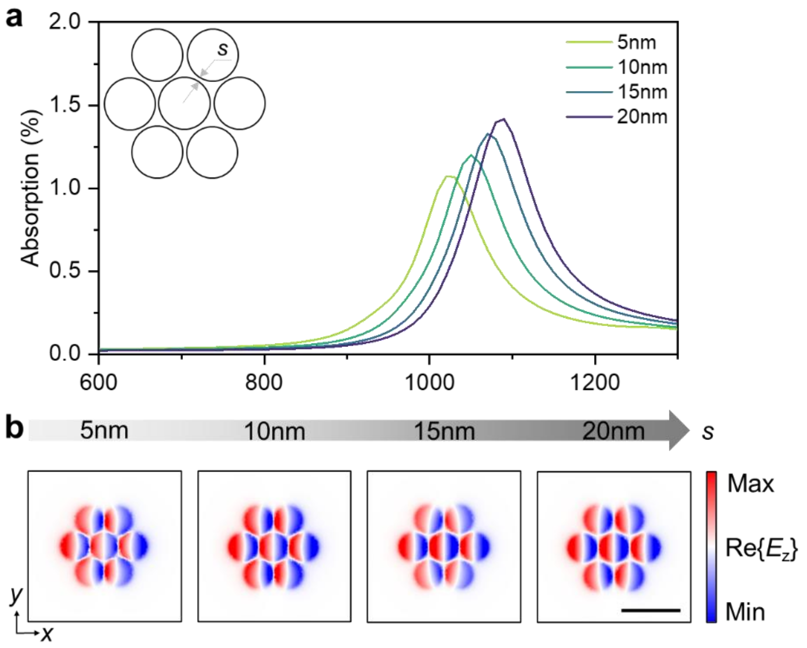

Supplementary Figure 4. Inter-disk spacing tuning in heptamer disks. (a) Simulated absorption spectra of the graphene heptamer disk array with varying edge-to-edge spacing (s = 5, 10, 15, 20 nm). **(b)** Spatial distribution of Re{E_z} at the D-mode resonance for each spacing, measured 10 nm above graphene. The scale bar represents 200 nm for all panels.

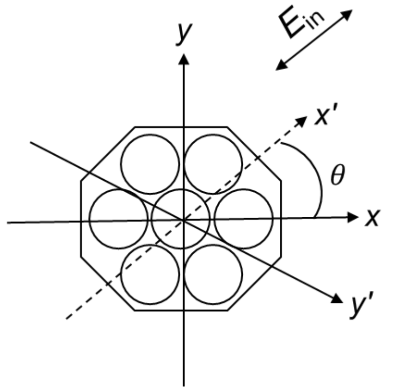

Supplementary Figure 5. Top-down view of the metasurface structure. Schematic shows the incident electric field (E_{in}) aligned with the x'-axis, which is rotated by an angle θ with respect to the primary axis (x-axis) of the structure.

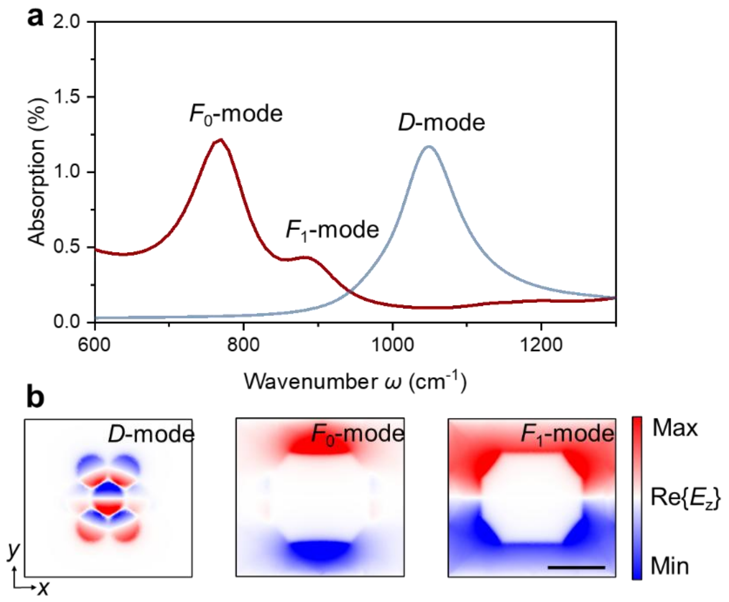

Supplementary Figure 6. Optical response of graphene plasmonic octagonal frame and heptamer disk under incident polarization angles of 90°. (a) Simulated absorption spectra of the graphene octagonal frame (red curve, F_0 and F_1 modes) and heptamer disks (blue curve, *D*-mode) under incident light polarized along the device's *y*-direction. The fundamental resonance frequencies of the *D*-mode, F_0 mode, and F_1 mode are similar to those observed under *x*-polarization (Figure 2a, b), confirming the polarization independence of their intrinsic mode energies. **(b)** Spatial distribution of the real part of the electric field z-component (Re{E_z}) for the *D*-mode (left), F_0 mode (middle), and F_1 mode (right) at 10 nm above the graphene surface. The scale bar represents 200 nm for all panels.

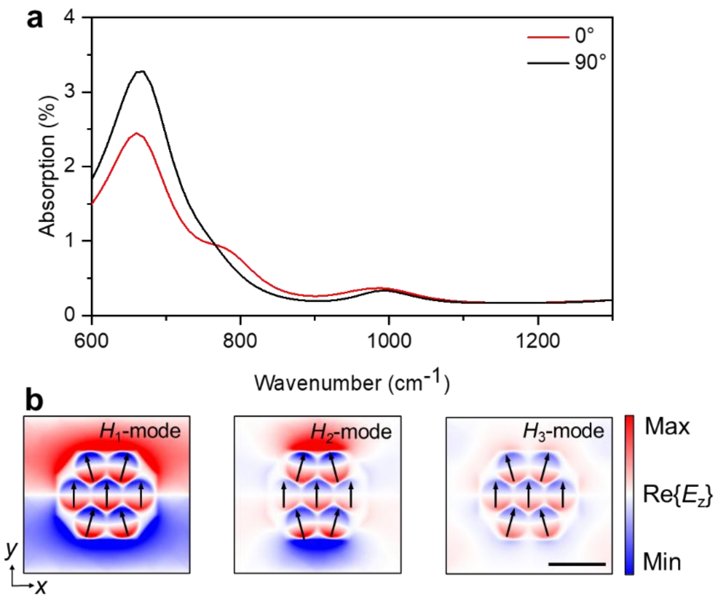

Supplementary Figure 7. Optical response of a graphene plasmonic octagonal frame with a heptamer disk under incident polarization angles of 0° and 90°. (a) Simulated absorption spectrum of the coupled octagonal frame-heptamer disk structure with incident light polarized at 90° and 0°. The H_2 mode absorption is fully suppressed under incident polarization angle of 90°, while H_1 and H_3 modes retain significant intensity. **(b)** Spatial distributions of Re{E_z} for modes H_1 (left), H_2 (middle), and H_3 (right) at resonance frequencies, measured 10 nm above graphene. The scale bar represents 200 nm for all panels.

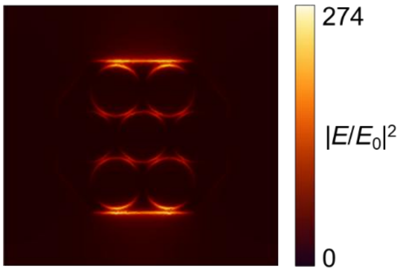

Supplementary Figure 8. Simulated electric near-field intensity enhancement. Simulated electric near-field intensity enhancement $|E/E_0|^2$ of H_2-mode under incident polarization angles of 90 °, where $|E_0|$ denotes the incident field amplitude.

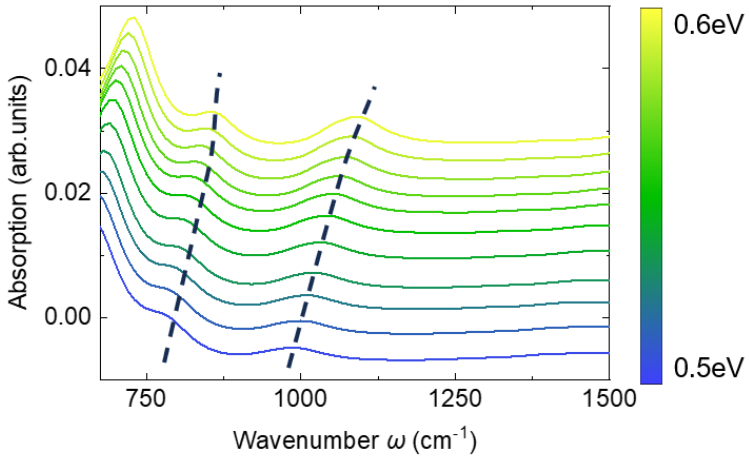

Supplementary Figure 9. Tuning graphene plasmons with graphene Fermi energy. Simulated absorption spectra of the graphene octagonal frame-heptamer disk coupled metamaterial as the Fermi energy (E_F) is finely tuned from 0.5 eV to 0.6 eV in increments of 0.01 eV. The color gradient (blue to yellow) corresponds to increasing E_F. The hybridized modes H_2 (left dashed line) and H_3 (right dashed line) exhibit continuous blue-shifting with elevated E_F, consistent with the experiments in Figure 4b.

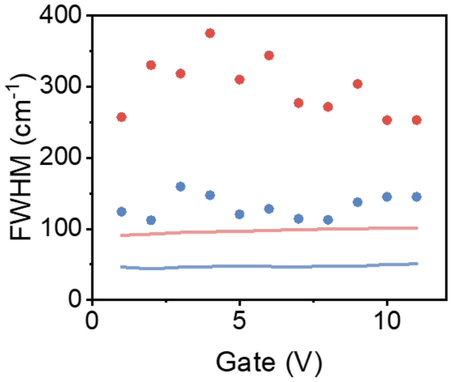

Supplementary Figure 10. Comparison of experimental and simulated linewidths. Full width at half maximum (FWHM) of H_2 (blue) and H_3 (red) extracted from experimental data (scattered dots) and simulations (solid lines). Both linewidths are relatively stable across a wide tuning range, indicating robust mode lifetimes that are largely independent of carrier concentration. The wider experimental FWHM is attributed to fabrication defects, which reduce carrier mobility and cause resonance broadening.

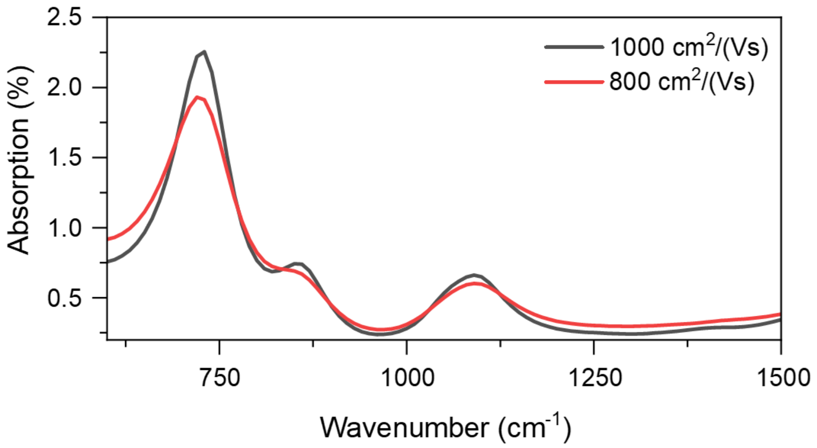

Supplementary Figure 11. Simulated absorption spectra for graphene plasmons at different carrier mobilities. The black and red lines correspond to carrier mobilities of 1000 cm²/(Vs) and 800 cm²/(Vs), respectively.
